# Plant species‐dependent transmission of *Escherichia coli* O157:H7 from the spermosphere to cotyledons and first leaves

**DOI:** 10.1111/1758-2229.13115

**Published:** 2022-08-15

**Authors:** Kathryn Mary Wright, Peter John Wright, Nicola Jean Holden

**Affiliations:** ^1^ The James Hutton Institute Invergowrie, Dundee UK; ^2^ Marine Scotland Science Aberdeen UK; ^3^ SRUC, Department of Rural Land Use, Craibstone Estate Aberdeen UK

## Abstract

The colonization of six edible plant species: alfalfa, broccoli, coriander, lettuce, parsley and rocket, by the human pathogen Shigatoxigenic *Escherichia coli* was investigated following two modes of artificial inoculation of seeds, by soaking or watering. The frequency and extent of colonization of cotyledons depended on the mode of inoculation, with three, rapidly germinating species being successfully colonized after overnight soaking, but slower germinating species requiring prolonged exposure to bacteria by watering of the surrounding growth media. Separate analysis of the cotyledons and leaves from individual plants highlighted that successful colonization of the true leaves was also species dependent. For three species, failure of transfer, or lack of nutrients or suitable microhabitat on the leaf surface resulted in infrequent bacterial colonization. Colonization of leaves was lower and generally in proportion to that in cotyledons, if present. The potential risks associated with consumption of leafy produce are discussed.

## INTRODUCTION

The food‐borne human pathogen Shigatoxigenic *Escherichia coli* (STEC) has the potential to cause a range of serious diseases including haemorrhagic colitis, haemolytic uraemic syndrome and central nervous system damage (Kaper et al., 2004). Recent outbreaks have been traced to contamination of fresh produce including Romaine lettuce (CDC, 2018a, 2018b, 2019) or leafy greens (CDC, 2017, 2020a). It is now recognized that STEC have adaptations in common with other phyllosphere bacteria that enhance their ability to colonize and survive on plants including adhesion to the cuticle, nutrient acquisition and tolerance to biotic and abiotic stress (Holden et al., 2009; Lemanceau et al., 2017; Lim et al., 2014; Méric et al., 2013).

During germination, the leakage of solutes and low‐molecular‐weight metabolites from the seed allows recruitment of microorganisms from inside the seed or from the surrounding environment to form the spermosphere and subsequently the rhizosphere (Lemanceau et al., 2017; Nelson, 2004; Schiltz et al., 2015). This nutrient release helps to explain why numerous STEC outbreaks have been associated with sprouted seeds including alfalfa (CDC, 2016), clover (CDC, 2012, 2014, 2020b), fenugreek (Buchholz et al., 2011) and white radish sprouts (Michino et al., 1999; Watanabe et al., 1999), the source of isolate STEC O157:H7 Sakai employed in the current study. Similarly, the young seedlings consumed as microgreens/microherbs present favourable environments for extensive growth of STEC (Işık et al., 2020; Wright & Holden, 2018; Xiao et al., 2014, 2015). For more mature plants, leaf age is known to be a factor that impacts colonization (Brandl & Amundson, 2008; Thompson et al., 1993).

Whilst germinating seeds can be readily colonized by human pathogenic bacteria including STEC, previous studies have not followed this colonization to the stage of leaf development (Cui et al., 2018). Investigation of broccoli microgreens grown on textile‐fibre matting suggested that the colonization of the leaves by STEC was greatly reduced compared to that on the cotyledons (Wright & Holden, 2018), raising questions about the impact of these two distinct developmental stages in bacterial colonization. To address this, we examined the colonization ability of STEC on six edible crop species, inoculated during germination and subsequently grown in compost. Colonization by STEC‐Sakai was examined by either detecting presence using an indicator medium or estimating infection level using an most probable number (MPN) method (Wright et al., 2021). For each plant species, both developmental stages were examined to inform on any preferential levels of colonization and/or transmission within the plant host. Two methods of inoculation were employed to compensate for different rates of seed germination and ensure the presence of STEC during the development of the spermosphere, with colonization being investigated at an individual plant and tissue level. We found that the frequency of, and the level of colonization of cotyledons and subsequent transfer to the true leaves is species dependent.

## EXPERIMENTAL PROCEDURES

### Bacteria

STEC isolate Sakai (kanamycin resistant) (Dahan et al., 2004), transformed with the reporter p*gyrA‐gfp* plasmid (Holden et al., 2006) (chloramphenicol resistant) for detection, was grown in defined medium with chloramphenicol (25 μg ml^−1^) at 18°C as described previously (Wright & Holden, 2018). Cultures were diluted to a cell density of OD = 0.2_600nm_ (~8.0 log_10_ CFU ml^−1^) in plant growth medium 0.5× Murashige and Skoog (MS. Murashige & Skoog, 1962) adjusted to pH 5.8 with NaOH (Duchefa product M0222) and further diluted as required.

### Plant material

**FIGURE 1:**
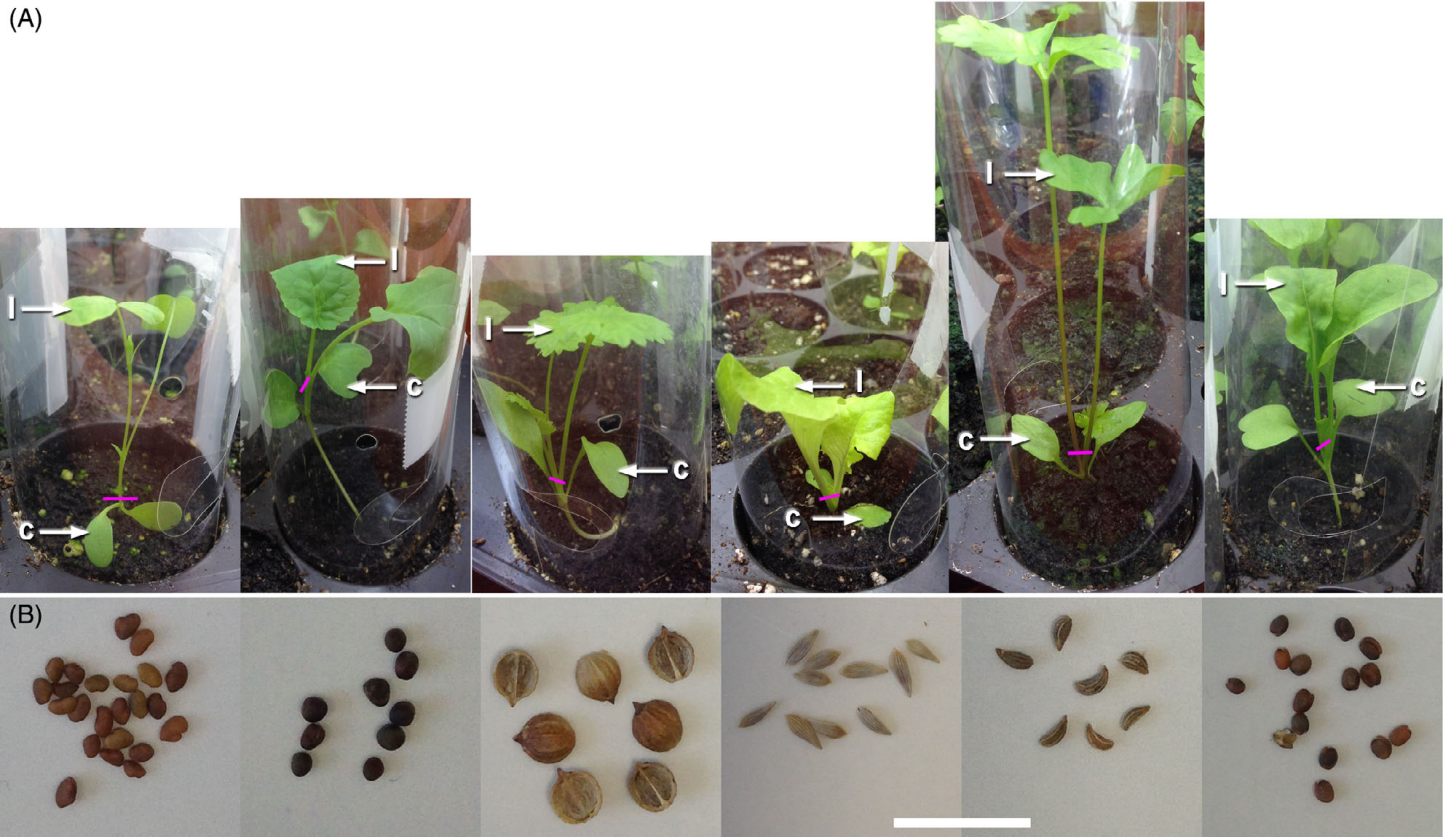
Plants and seeds. (A) Plants of alfalfa, broccoli, coriander, lettuce parsley and rocket within plastic collars recorded immediately prior to harvest, indicating the approximate point of division (magenta line) between cotyledon (c) and leaf (l) samples. The diameter of each compost‐containing cavity is 5 cm to provide an estimate of scale. (B) Seeds of each species (scale bar = 1 cm)

Seed stocks, as detailed in Table 1, were purchased from Dobies, Paignton, UK; Unwins, Huntingdon, UK; Chiltern Seeds, Wallingford, UK. Seeds (see Figure 1 for seed morphology) were surface sterilized in 5% domestic bleach solution (Domestos, Unilever: includes 10% sodium hypochlorite, 0.1%–1% sodium hydroxide and surfactant) for 5 min followed by six rinses in sterile distilled water (SDW). For inoculation, seeds were either ‘soaked’ overnight in ½ MS containing STEC‐Sakai at 10^7^ CFU ml^−1^ and then rinsed 6× in SDW. Alternatively, seeds were transferred to plastic tubs lined with purple matting and ‘watered’ with one dose of 15 ml ½ MS containing GFP‐Sakai at 10^3^ CFU ml^−1^ (see Wright & Holden, 2018), as indicated (Table 2) and maintained in a growth cabinet (16 h light, 8 h dark, 21°C) until germinated and showing signs of cotyledon and root emergence (4–11 days). Whether soaked or watered, germinated seedlings were transferred to ‘Araflat’ trays containing commercial compost, where necessary fitted with plastic collars to prevent cross contamination between plants (Arasystem, Ghent, Belgium) and maintained in a quarantine growth room (16 h day, 23°C, 8 h night, 21.5°C, humidity 60%–80%, watering with mains tap water).

**TABLE 1:** Species used in this study detailing common, species and family names, and seed supplier

Name	Species	Family	Supplier
Alfalfa	*Medicago sativa*	*Fabaceae*	Dobies
Broccoli	*Brassica oleracea*	*Brassicaceae*	Unwins
Coriander	*Coriandrum sativum*	*Apiaceae*	Unwins
Parsley (Italian Plain Leaved)	*Petroselinum crispum*	*Apiaceae*	Unwins
Rocket, Victoria	*Eruca vesicaria subsp. Sativa*	*Brassicaceae*	Unwins
Lettuce, Curled and Oak leaf, ‘Lollo‐Rossa’	*Lactuca sativa*	*Asteraceae*	Chiltern

For the presence or absence assessment, plants were harvested following leaf emergence (Figure 1) using forceps and scalpel treated in 70% ethanol and separated into the cotyledons including the stem cut just above soil level, or the leaves including the stem cut above the cotyledons. Samples were collected in 2 ml or 5 ml tubes and MacConkey purple broth plus chloramphenicol (final 25 μg ml^−1^) (MAC‐p‐cml) added. For quantification, samples were harvested as above into pre‐weighed tubes, weighed, ground and resuspended in 1 ml PBS. These extracts were serially diluted 1:10 in PBS, with five steps for the cotyledons and three steps for the leaves, subsampled in triplicate with 100 μl added to 400 μl MAC‐p‐cml and incubated for 40 h (Wright et al., 2021). The addition of chloramphenicol selected for the presence of STEC‐Sakai, which was scored as the change in colour from purple to yellow. MPN of bacteria was estimated using the method of Jarvis et al. (2010). For all‐negative samples, the value of 1 was recorded as below the limit of detection (=3). For samples with all‐positive results, which should represent infinity, the next lowest value for 3‐3‐2 was recorded and multiplied up as appropriate. For each species, the extent of STEC colonization was investigated under inoculation conditions, which resulted in frequent colonization of the cotyledons.

The time to germination was assessed by sowing five replicate plates, containing 0.5% distilled water agar, with 10 seeds of each species per plate. The plates were maintained at room temperature and the emergence of radicles scored daily.

### Statistical analysis

All statistical analyses were conducted in R version 3.6.3. For those species and inoculation method where prevalence was >0 and <1, quantitative differences in the proportion of cotyledons and leaves infected among species and method of inoculation was estimated using a logistic model followed by an odds ratios test for pairwise comparisons. For lettuce, this method was also used to compare between the two substrates. These logistic models were implemented using the ‘mgcv’, ‘car’ and ‘lsmeans’ packages. Non‐parametric tests were required for colonization rate determination (MPN g^−1^ FW) as the response variable did not conform to normality and homogeneity assumptions regardless of transformation. To test whether cotyledon and leaf colonization were related, a Spearman rank correlation was used. Differences in colonization rate were compared among species and inoculation method using a Kruskal–Wallis test and pairwise comparisons made using a Dunn's multiple comparison test with the Holm–Šidák method, implemented using the ‘dunns.test’ package.

## RESULTS

### The influence of species type and inoculation method on STEC seedling colonization

To determine any species‐dependent effect, the frequency of STEC‐Sakai colonization was assessed for six plant species (alfalfa, broccoli, coriander, lettuce, parsley, rocket) inoculated by ‘seed‐soaking’. Alfalfa showed the highest, and lettuce and parsley the lowest proportions of seedlings colonized (Table 2). Although STEC‐Sakai were isolated from the cotyledons of most species, there were differences in the proportion of plants with colonized leaves, with no bacteria being recovered from the leaves of coriander, lettuce or parsley. Similarly, only a low proportion of leaf colonization was observed in broccoli and rocket. In contrast, in alfalfa, the cotyledons of all plants were colonized along with a high proportion (0.79) of their leaves.

**TABLE 2:** Comparison of the proportion of plants with positive detections of STEC‐Sakai, for cotyledons and leaves

Species	Inoculation mode: Exposure time to STEC‐Sakai	*n*	Cotyledons		Leaves	
Alfalfa	Soak/short	33	1.00		0.79	A
Broccoli	Soak/short	79	0.61	A	0.08	B
Coriander	Soak/short	30	0.47	A	0.00	
Lettuce	Soak/short	95	0.07	B	0.00	
Parsley	Soak/short	25	0.12	B	0.00	
Rocket	Soak/short	50	0.70	A	0.16	B
Broccoli	Watered/long	10	1.00		0.10	B
Coriander	Watered/long	37	1.00		0.76	A
Lettuce	Watered/long	24	0.92	A	0.13	B
Parsley	Watered/long	25	0.92	A	0.48	A

*Note*: The number of plants per species and inoculation method is indicated. Letters refer to column wise proportions with no significant difference based on odds ratio test. Coloured boxes indicate level of colonisation, for maximum (purple), high (green) or low (blue) levels.

For the species that showed a low frequency of colonization after seed‐soaking (broccoli, coriander, lettuce, parsley), a prolonged exposure to STEC‐Sakai was applied by germination of seeds on matting ‘watered’ with the bacteria, for which we previously showed long‐term persistence of STEC‐Sakai (Wright & Holden, 2018). The frequency of STEC‐Sakai detection in the cotyledons was significantly higher using this alternative inoculation regime. For coriander and parsley, this resulted in a high proportion of plants with colonized leaves (Table 2). However, the proportion of broccoli and lettuce with colonized leaves remained low.

Since there was a possibility that the proportion of colonization was related to germination of the different species, their time to germination was compared. In general, the species fell into two classes of relatively rapid germination, where seeds germinated either within 3–4 days of sowing for alfalfa, broccoli, lettuce and rocket or 8–17 days after sowing, for coriander and parsley.

### STEC populations of cotyledons and leaves

**FIGURE 2:**
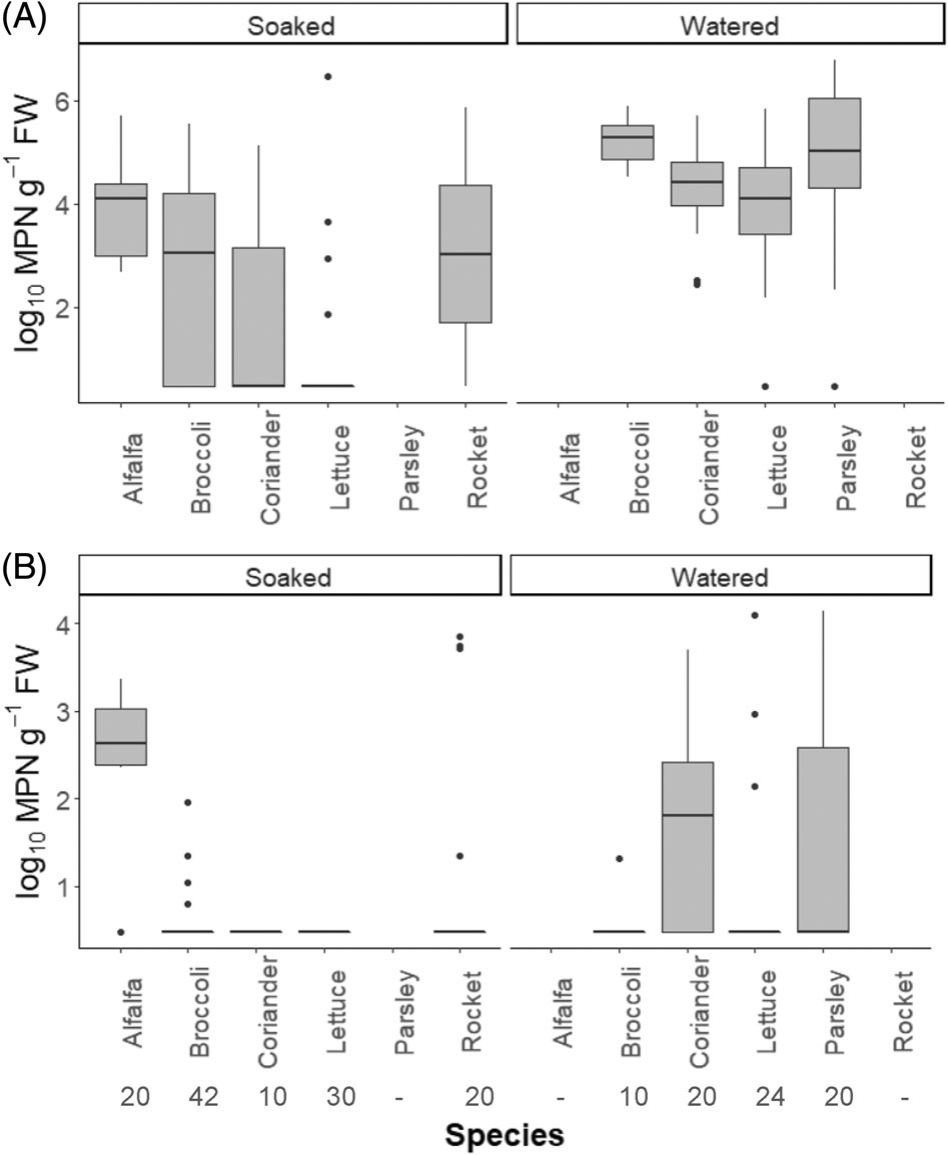
Boxplot of median estimates of bacterial numbers recovered from (A) cotyledon, or (B) leaf expressed as log_10_ MPN g^−1^ FW with 25 and 75% quartile ranges and 95% confidence intervals for soaked and watered plant species. The number of plants analysed is shown below the *x*‐axis.

To quantify the extent of colonization, the level of STEC‐Sakai was estimated using the MPN technique. The colonization rate of STEC‐Sakai varied between species and inoculation methods for both cotyledons (Figure 2A) and leaves (Figure 2B, Kruskal–Wallis *χ* ^2^_8_ = 83.1; *p* < 0.001). Estimates of the number of STEC g^−1^ FW in colonized cotyledons ranged from log_10_ 1.1 to 6.7 with many plants having in excess of log_10_ 3.0 g^−1^ FW (Figure 2A). Watering‐inoculated plants had a significantly higher log MPN than soaked (*p* > 0.05). For seed soaked‐inoculation, alfalfa (*Z* = 4.595; *p* = 0.0001), broccoli (*Z* = 3.275; *p* = 0.013) and rocket (*Z* = 3.475; *p* = 0.007) had a significantly higher MPN than lettuce. For watering‐inoculation, there was no significant difference among broccoli, coriander, lettuce and parsley (*p* > 0.1).

In colonized leaves, estimates of the number of STEC g^−1^ FW ranged from log_10_ 0.8 to 4.1 (Figure 2B). Seed‐soaked alfalfa had a significantly higher MPN than broccoli, coriander and rocket (*p* < 0.001) and no bacteria were detected in lettuce (Table 2). For coriander, inoculation by watering resulted in a significantly higher estimate of STEC‐Sakai than inoculation by seed soaking (*Z* = 3.168, *p* = 0.020). There were no significant differences between the remaining plant species for the watering‐inoculation treatment, despite a large range of MPN estimates for watering‐inoculated parsley.

**FIGURE 3:**
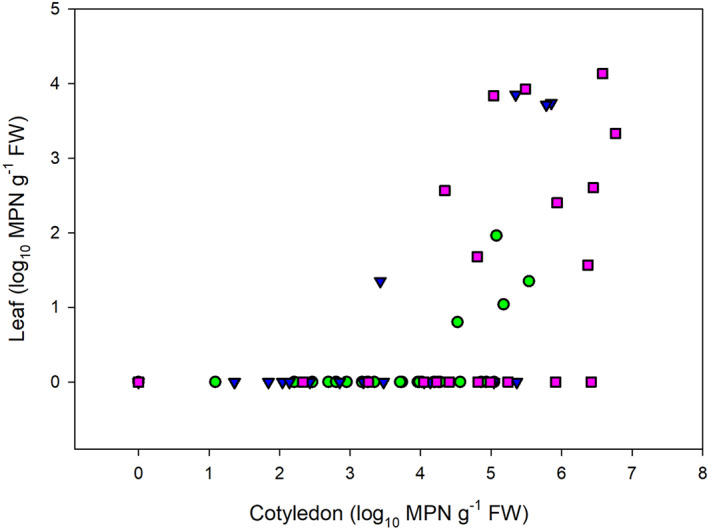
Relationship between leaf and cotyledon colonization (log_10_ MPN g^−1^ FW) for broccoli (circle), rocket (triangle) and parsley (square)

Detection of bacteria in true leaves only occurred with positive detection in the associated cotyledons and the level of colonization was always higher in cotyledons than leaves of a given plant. For seed‐soaked broccoli and rocket and watered parsley the level of colonization in leaves positively correlated with that of the cotyledons (*R*s > 0.49; *p* < 0.01; Figure 3).

## DISCUSSION

This study addressed two main questions: the likelihood of colonization of seedlings by STEC‐Sakai following exposure during germination and the frequency of colonization of the leaves. The likelihood that a seedling was colonized depended on the duration of exposure and species. For alfalfa, broccoli and rocket, a short, overnight, exposure to STEC (i.e. seed soaking) was sufficient to result in frequent plant colonization. This may be explained by rapid uptake of liquid into the seed and early germination, since the seed coat of these species was frequently observed to have fractured during overnight seed soaking and seeds germinated within 3–4 days. The mode of inoculation is likely to directly impact on the bacterial physiological stress response, with respect to nutrient availability and acquisition, and access to the emerging radical. Although adhesion of bacteria to the outside of the seed coat could be expected following overnight treatment with high concentrations of STEC, for coriander, lettuce and parsley, either this did not occur or the bacteria did not survive until seed germination. Parsley and coriander seeds took longer to germinate and appear to have more impermeable seed coats. Slower germination has previously been implicated to enhance the epiphytic colonization of spring wheat by *Pseudomonas syringae* (Fryda & Otta, 1978), but this does not appear to be the case in the current study. In lettuce, although germination was rapid, other factors possibly including competition with soil‐borne microbiota, the presence of inhibitory compounds or a lack of nutrients due to the slower release of sugars during germination appear to be involved. A similar absence of colonization by *E. coli* 0157:H7 of bulked microgreen samples of lettuce grown in peat has been observed, although colonization was observed when grown in perlite (Işık et al., 2020). The ability of STEC to survive and divide under low nutrient levels in the absence of competition has been previously demonstrated (Wright & Holden, 2018) and this would enable survival of STEC in watered matting to allow the observed enhanced colonization of the slower germinating coriander and parsley.

The second question addressed the frequency of colonization of the leaves particularly following inoculation under conditions resulting in frequent colonization of the cotyledons. For three species: broccoli, lettuce and rocket, this was infrequent (around 15% of plants), whereas around half of parsley plants and over 75% of alfalfa and coriander plants had colonized leaves. These differences were confirmed when colonization of the cotyledons or leaves was quantified at an individual plant level. Previous studies have demonstrated differences in the frequency of internalization, rather than total colonization, of STEC by different plant species following inoculation via contaminated water, but the use of bulked material did not allow separate analysis of cotyledons and leaves (Chitarra et al., 2014). Plant specific, quantitative differences have also been observed in the colonization of sprouting seeds, with alfalfa and lettuce having significantly higher numbers of EHEC cells, followed by fenugreek and tomato seedlings, but this was not followed to the stage of leaf development (Cui et al., 2018).

Due to the large variation between samples, MacConkey purple broth was used in a quantitative MPN method to estimate the total level of epiphytic and potentially endophytic STEC colonization. The errors inherent in an MPN design of three replicates of three sequential 10‐fold dilution levels, mean that this is an estimate of the magnitude of the density rather than a more accurate quantification (ISO 7218, 2007). As MPN estimates are not a continuous variable we treated these values as ordinal ranks in non‐parametric tests. The results confirm that the populations of STEC are highly variable as observed previously for population of epiphytic bacteria ranging from non‐detected to 10^6^ CFU/plant part (Hirano & Upper, 1983) and colonization of the cotyledons is much higher than the leaves. Whilst the initial colonization of the cotyledons may be supported by exudates released during the early stages of germination (Nelson, 2004; Schiltz et al., 2015), further bacterial growth may depend on carbon leached onto the tissue surface (Andrews & Harris, 2000). This nutrient source will be reduced in leaves, which develop thicker cuticles (Hunter et al., 2010; Remus‐Emsermann et al., 2011).

The mechanism by which STEC‐Sakai migrate from the germinating seed to the cotyledons or leaves is unclear. Studies of epiphytic bacteria have suggested that some bacteria are motile and are dispersed by swimming whilst others may be established on the growing point and divide on the growing tissue (Hirano & Upper, 1983; Leben, 1965). Previous studies have shown migration of GFP‐labelled *E. coli* O157:H7 from the crown to flowers of *Arabidopsis thaliana*, but it is unclear whether this motility involved functional flagella as observed for *Salmonella enterica* (Cooley et al., 2003). The low frequency and levels of leaf colonization by STEC‐Sakai observed for broccoli, lettuce and rocket may be the result of failure to migrate to the expanding leaves or failure to establish and survive on the leaf surface following transfer during shoot and leaf expansion. Further, the phyllosphere may be a hostile environment for bacteria due to stresses including desiccation, UV radiation and nutrient availability (O'Brien & Lindow, 1989; Remus‐Emsermann & Schlechter, 2018) and movement of bacteria has often been associated with high rather than low humidity levels (Fryda & Otta, 1978; Hirano & Upper, 1983; Lindow, 1991). Other factors, including competition with other endophytic bacteria, or the release of toxic compounds by the plant may also influence the survival of STEC on leaves (Aruscavage et al., 2006). The higher biofilm and extracellular matrix production displayed by *E. coli* isolates from plants are likely adaptations to mitigate against these environmental conditions (Méric et al., 2013). The frequent and higher levels of colonization of leaves of alfalfa, coriander and parsley grown under similar levels of humidity suggest that nutrient availability or leaf surface topology may be more suited to support STEC growth in these species (Doan & Leveau, 2015).

Our results highlight that the cotyledons of seedlings germinating in the presence of STEC are likely to be contaminated and therefore edible foods that include the cotyledons, for example microgreens and baby‐leaf crops, exposed to contaminated water present an increased food safety risk. For alfalfa, coriander and parsley, this risk may also be extended to consumption of the leaves. However, for broccoli, lettuce and rocket, the low frequency and extent of leaf colonization is consistent with previous findings implicating overhead irrigation as a major source of contamination of older plants (Solomon et al., 2002). Importantly, we have shown an interaction between the plant species, mode of inoculation, which represents transmission pathway, and developmental age, highlighting the need to consider each plant crop system independently.

## CONFLICT OF INTEREST

The author declares that there is no conflict of interest that could be perceived as prejudicing the impartiality of the research reported

## Data Availability

The data that support the findings of this study are available from the corresponding author upon reasonable request.
